# Atovaquone and quinine anti-malarials inhibit ATP binding cassette transporter activity

**DOI:** 10.1186/1475-2875-13-359

**Published:** 2014-09-13

**Authors:** Sanna R Rijpma, Jeroen JMW van den Heuvel, Maarten van der Velden, Robert W Sauerwein, Frans GM Russel, Jan B Koenderink

**Affiliations:** Department of Pharmacology and Toxicology, Radboud University Medical Centre, Nijmegen, Netherlands; Department of Medical Microbiology, Radboud University Medical Center, Nijmegen, Netherlands

**Keywords:** ABC transporter, P-glycoprotein, BCRP, BSEP, MRP, Anti-malarial, Transport, Vesicle

## Abstract

**Background:**

Therapeutic blood plasma concentrations of anti-malarial drugs are essential for successful treatment. Pharmacokinetics of pharmaceutical compounds are dependent of adsorption, distribution, metabolism, and excretion. ATP binding cassette (ABC) transport proteins are particularly involved in drug deposition, as they are located at membranes of many uptake and excretory organs and at protective barriers, where they export endogenous and xenobiotic compounds, including pharmaceuticals. In this study, a panel of well-established anti-malarial drugs which may affect drug plasma concentrations was tested for interactions with human ABC transport proteins.

**Methods:**

The interaction of chloroquine, quinine, artemisinin, mefloquine, lumefantrine, atovaquone, dihydroartemisinin and proguanil, with transport activity of P-glycoprotein (P-gp), breast cancer resistance protein (BCRP), bile salt export pump (BSEP) and multidrug resistance-associated proteins (MRP) 1–4 were analysed. The effect of the anti-malarials on the ATP-dependent uptake of radio-labelled substrates was measured in membrane vesicles isolated from HEK293 cells overexpressing the ABC transport proteins.

**Results:**

A strong and previously undescribed inhibition of BCRP-mediated transport by atovaquone with a 50% inhibitory concentration (IC_50_) of 0.23 μM (95% CI 0.17-0.29 μM) and inhibition of P-gp-mediated transport by quinine with an IC_50_ of 6.8 μM (95% CI 5.9-7.8 μM) was observed. Furthermore, chloroquine and mefloquine were found to significantly inhibit P-gp-mediated transport. BCRP transport activity was significantly inhibited by all anti-malarials tested, whereas BSEP-mediated transport was not inhibited by any of the compounds. Both MRP1- and MRP3-mediated transport were significantly inhibited by mefloquine.

**Conclusions:**

Atovaquone and quinine significantly inhibit BCRP- and P-gp- mediated transport at concentrations within the clinically relevant prophylactic and therapeutic range. Co-administration of these established anti-malarials with drugs that are BCRP or P-gp substrates may potentially lead to drug-drug interactions.

## Background

ATP binding cassette (ABC) transporters are membrane-bound proteins that allocate a wide variety of compounds at the expense of ATP, even against steep concentration gradients [1]. P-glycoprotein (P-gp/ABCB1), bile salt export pump (BSEP/ABCB11), multidrug resistance-associated proteins (MRP1-4/ABCC1-4), and breast cancer resistance protein (BCRP/ABCG2) are among the most important drug transporters of the ABC protein family. ABC transport proteins are known for their capacity to protect the organism from potentially toxic xenobiotics through excretion, thereby decreasing intracellular concentrations. Indeed, typical localization of these export transporters are at the blood–brain barrier, placenta, gut, and at the apical side of liver and kidney cells. Two compounds may interact with the same transport protein through induction of expression, inhibition of protein function or competition of substrates. Pharmacokinetics of co-administered drugs can be critically altered when drug-drug interactions occur at the level of the ABC transport proteins, as distribution and selective excretion of these compounds may depend heavily on ABC protein-mediated transport. This can be reflected either in unexpected high blood plasma concentrations potentially causing toxic effects, or subtherapeutic concentrations at the site of action, diminishing therapeutic effects.

It is essential to assure effective blood plasma concentrations upon treatment with anti-malarial compounds in order to cure severely ill patients and prevent resistance acquisition through exposure of the parasite to sublethal blood plasma concentrations. The first-line treatment as recommended by the World Health Organization (WHO) currently consists of artemisinin-based combination therapy [2]. However, resistance against these regimens has been detected and the number of anti-malarials that can be subsequently applied are limited [3]. Toxic effects by unintended elevated blood plasma concentrations, however, should also be avoided.

Direct interaction with ABC transporter capacity of anti-malarial compounds has not been explored in detail. *In vitro* assays have indicated a possible effect on P-gp-mediated transport or expression after exposure to chloroquine, quinine, mefloquine, primaquine, amodiaquine, piperaquine, artemisinin, and dihydroartemisinin, however, contradictory conclusions concerning the interaction of anti-malarial compounds with ABC transport proteins could be drawn from different experimental set-ups [4–9]. A possible interaction of anti-malarial compounds with MRP-type transporters and BCRP has also been described [10–13]. Co-administration of anti-malarial compounds with other drug types is highly anticipated. For instance, human immunodeficiency virus (HIV) and malaria co-infections are likely to occur, as there is a high overlap in geographical dissemination [14]. Therefore, the effect of anti-malarial compounds on ABC-mediated transport capacity should be explored in more detail in order to secure the most effective treatment strategies for patients receiving multiple drug regimens.

In this study the direct interaction of a panel of eight well-known anti-malarial compounds (chloroquine, quinine, artemisinin, mefloquine, lumefantrine, atovaquone, dihydroartemisinin, and proguanil) with transport activity of P-gp, MRP1-4, BCRP and BSEP in a vesicular overexpression transport assay have been analysed. Anti-malarials (100 μM) that caused a decrease in substrate transport larger than 66.7% were further characterized to determine their 50% inhibitory concentrations (IC_50_). Potent and previously undescribed inhibition of BCRP-mediated transport by atovaquone and P-gp-mediated transport by quinine was observed at concentrations within their therapeutic range.

## Methods

### Materials

[6,7-^3^H(*N*)]Estrone-sulphate ammonium salt ([^3^H]-E1S, specific activity 45.6 Ci/mmol), Tauro[carbonyl-3H]Cholic Acid sodium salt ([^3^H]TCA) (5 Ci/mmol) and [6,7-^3^H(*N*)]Estradiol 17-β-D-glucuronide ([^3^H]-E_2_17βG) (34.3 Ci/mmol) were purchased from PerkinElmer Life and Analytical Sciences (Groningen, Netherlands). [^3^H(*N*)]-methyl quinidine ([^3^H]-NMQ) (80 Ci/mmol) and unlabelled NMQ [N-methyl-quinidine] were purchased from Solvo Biotechnology (Szeged, Hungary). Bac-to-Bac and Gateway systems, Dulbecco’s modified Eagle’s medium, GlutaMAX-I culture medium, and foetal calf serum were purchased from Life Technologies (Bleiswijk, Netherlands). Primers were purchased from Biolegio (Nijmegen, Netherlands), and a plasmid purification midiprep kit was from Genomed (Löhn, Germany). Triple flasks (500 cm^2^) were purchased from Sanbio BV Biological Products (Uden, Netherlands). Estradiol 17-β-D-glucuronide (E_2_17βG), estrone-sulphate (E1S), taurocholic acid (TCA) adenosine 5’-triphosphate magnesium salt (bacterial source), goat-anti-mouse IgG antibody IRDye 800 and goat-anti-rabbit Alexa 680 secondary antibodies, chloroquine (CQ), quinine (Q), artemisinin (ART), mefloquine (MQ), lumefantrine (L), atovaquone (ATO), dihydroartemisinin (DHA), and proguanil (PG) were purchased from Sigma-Aldrich (Zwijndrecht, Netherlands). Protein concentrations were determined with a Bio-Rad protein assay kit from Bio-Rad Laboratories (Veenendaal, Netherlands), and 96-well filter plates were purchased from Millipore (Etten-leur, Netherlands).

### Baculovirus generation

Human P-gp, BCRP, BSEP and MRP1-4 had previously been cloned into the Gateway pDONR221 vector. Sequences matched accession numbers NM_000927, NM_004827, NM_003742, NM_004996, NM_000392, NM_00378, and NM_005845 respectively [15–19]. Some sequences did hold silent mutations of described polymorphisms. Gateway cloning was used to transfer the genes into a VSV-G improved pFastBacDual vector for mammalian cell transduction. The production of baculovirus was executed according to the Invitrogen Bac-to-Bac manual.

### Cell culture and transduction

HEK293 cells were grown to 40% confluency in Dulbecco’s modified Eagle’s medium-GlutaMAX-I containing 10% foetal calf serum at 5% CO_2_ in 500 cm^2^ triple flasks. Culture medium was removed and 25 mL of medium combined with 10 mL virus was added and incubated at RT for 20 min, followed by the addition of another 40 mL of complete medium including 5 mM sodium butyrate to enhance protein expression.

### Membrane vesicle isolation and protein analysis

Cells were harvested three days post transduction by a 5-min centrifugation step at 3,000 *g*. Cells were resuspended in ice-cold hypotonic buffer (0.5 mM sodium phosphate, 0.1 mM EDTA, pH 7.0) containing protease inhibitors (100 mM phenylmethylsulfonyl fluoride, 5 mg/ml aprotinin, 5 mg/ml leupeptin, 1 mg/ml pepstatin and 1 mg/ml E-64) and shaken at 4°C for 30 min. This lysate was centrifuged 100,000 *xg* for 30 min at 4°C, after which the pellet was homogenized in ice-cold TS buffer (10 mM Tris-HEPES and 250 mM sucrose, pH 7.4) supplemented with protease inhibitors described before using a tight-fitting Dounce homogenizer for 25 strokes. Two subsequent centrifugation steps at 4°C of firstly 20 min at 4,000 *g* followed by supernatant centrifugation for 60 min at 100,000 g ensured harvesting of the membrane fraction. The pellet was resuspended in ice-cold protease free TS buffer and passed 25 times through a 27-gauge needle to enhance membrane vesicle formation. Protein concentration in these vesicles was determined using the Bio-Rad protein assay, vesicles were flash-frozen in N_2_ and stored at -80°C.

### Vesicular transport assays

A rapid filtration technique that has been described earlier was applied to evaluate uptake of transporter specific substrates into the vesicles; NMQ for P-gp, E1S for BCRP, E_2_17βG for MRP1-4 and TCA for BSEP [20]. Briefly, 0.015-0.15 μCi of labelled substrate was combined with unlabelled substrates to a concentration of 0.1-1 μM in a 30 μL reaction mixture with 4 mM ATP, 10 mM MgCl_2_ and 7.5 μg total protein membrane vesicles in TS buffer. Transport was allowed by transfer of the plates to 37°C during 1–5 min, a time-point within the linear phase of time-dependent transport, as previously determined [15–19]. Hereafter, the reaction was rapidly stopped by placing the plates back on ice and the addition of 150 μL ice-cold TS buffer. Samples were subsequently transferred to a 96-well filter plate that had been pre-incubated with TS buffer, and filtered using a multiscreen HTS-vacuum manifold filtration device (Millipore). Filters were washed and extracted, after which 2 mL scintillation fluid was added to each filter. Radioactive signal on the filters was determined by liquid scintillation counting. Negative controls included eYFP-transduced vesicles and AMP instead of ATP in the reaction mixture.

In the first screen, all anti-malarial compounds were added to the reaction mixture to evaluate transport inhibition at a concentration of 100 μM. Solvents were used as negative controls, as CQ was dissolved in milliQ, Q and ART in methanol, MQ, L, ATO and DHA in DMSO and PG in 50% ethanol. When ATP-dependent uptake was reduced more than 66.7%, the compound was considered a potential inhibitor, and multiple concentrations were tested in the reaction mixture to determine the IC_50_ value. All concentrations were tested in duplicates or triplicates in two individual biological replicates containing vesicles of independent transductions. Results were depicted and statistically analysed using Graphpad Prism, version 5.03. IC_50_ values were determined by nonlinear regression analysis of (log) inhibitor-response curves with variable slope. Maximal transport was restricted to 100%, and the minimum was set to be equal or greater than 0%. Statistical analysis was performed using IBM SPSS Statistics 20, applying one-way ANOVA (Analysis of variance).

## Results

### Inhibitory profile of anti-malarials against ABC transporter activity

The inhibitory characteristics against the ABC transporters of eight well-known anti-malarials; CQ, Q, ART, MQ, L, ATO, DHA and PG, was investigated at a 100 μM concentration. For each transporter protein, specific radio-labelled substrates were applied to measure ATP-dependent transport into the vesicular overexpression system; N-methyl quinidine (7nM radio-labelled diluted with 90 nM non-radio-labelled) for P-gp, estrone sulphate (74 nM) for BCRP, estradiol 17-β-D glucuronide (150 nM) for MRP1-4 and taurocholic acid (1 μM) for BSEP [16–19].

**Figure 1:**
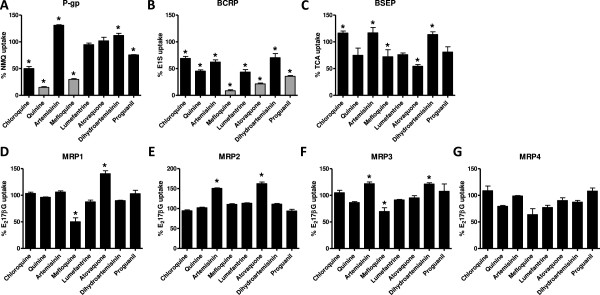
**Inhibitory effect of anti-malarial drugs on ABC transport activity.** The inhibitory effect of 100 μM of CQ, Q, ART, MQ, L, ATO, DHA and PG on ABC transporter activity was assessed. Transport was measured in pmol/mg protein/min and expressed as percentage of solvent controls, which represent 100% transport. Bars with * are significantly different from solvent controls, p < 0.05. **A** P-gp-mediated transport of NMQ was significantly inhibited by CQ, Q, MQ and PG, and increased by ART and DHA. **B** BCRP-mediated transport of E1S was significantly inhibited by all compounds, most pronounced inhibitors were MQ and ATO. **C** BSEP-mediated transport of TCA was significantly inhibited by ATO, but not by the other anti-malarials. Induction of transport was observed for CQ, ART and DHA. **D-G** MRP1-4-mediated E217βG transport. MQ significantly inhibited MRP1 and MRP3 transport activity. Furthermore, induction of MRP1 mediated transport was found for ATO, which, together with ART, also stimulated MRP2 transport activity. MRP3 mediated transport was stimulated by both ART and DHA. Inhibition larger than 66.7% was found for Q and MQ on P-gp transport, as well as MQ, ATO and PG on BCRP transport activity (highlighted bars).

A significant inhibitory effect of 100 μM CQ, Q, MQ and PG on P-gp-mediated NMQ transport was observed. CQ reduced NMQ transport to 50% (p < 0.001) and PG to 76% (p < 0.001), whereas Q and MQ gave more pronounced inhibitory effects to 15% (p < 0.001) and 30% (p < 0.001) P-gp-mediated NMQ transport, respectively. ART and DHA slightly induced transport activity to 131% (p < 0.001) and 112% (p = 0.033), respectively (Figure 1A). All anti-malarials inhibited BCRP-mediated estrone sulphate transport activity at 100 μM concentrations. Most potent inhibitors were MQ, ATO and PG, which reduced estrone sulphate transport to 8.5%, 22% and 36% with p < 0.001, respectively (Figure 1B). CQ reduced transport to 69%, Q to 45%, ART to 62%, L to 44%, and DHA to 70% of solvent-exposed BCRP-mediated transport capacity (p < 0.001). Significant inhibition of taurocholic acid transport by BSEP was observed for ATO, which reduced uptake to 54% (p < 0.001) and MQ, which reduced uptake to 72% (p = 0.037). Furthermore, induction of BSEP transport activity was found for CQ (117%, p < 0.001), ART (117%, p < 0.001) and DHA (114%, p < 0.001) (Figure 1C). MQ was found to have a modest but significant inhibitory effect on estradiol 17-β-D glucuronide transport by MRP1 as this was reduced to 50% (p < 0.001), whereas ATO was observed to induce this process to 141% (p < 0.001) (Figure 1D). Induction was also observed for ART and ATO on MRP2-mediated estradiol 17-β-D glucuronide transport to 151% (p = 0.015) and 162% (p = 0.020), respectively. However, no significant inhibition was measured for any of the anti-malarials tested (Figure 1E). MRP3-mediated translocation of estradiol 17-β-D glucuronide was significantly inhibited by MQ at a 100 μM concentration to 70% (p = 0.001), whereas ART and DHA induced this process to 122% (p = 0.016) and 121% (p = 0.020), respectively (Figure 1F). No significant estradiol 17-β-D glucuronide transport inhibition of MRP4 could be detected (Figure 1F). As the 100 μM concentration is not within the physiological range of compound exposure, the most potent inhibitors were selected for further investigation. Inhibition of Q and MQ on P-gp-mediated transport, as well as BCRP inhibition by MQ, ATO and PG, were studied in more detail to determine their potencies.

### Determination of inhibitory potency of strong inhibitors

Subsequently, transport inhibition assays were performed for a larger concentration range of Q, MQ, ATO and PG to evaluate P-gp or BCRP activity. Inhibition of transport was measured in a similar fashion applying the same specific radio-labelled substrates. Drug concentrations were logarithmically depicted, and a sigmoidal, inhibitor-response, variable slope equation was fitted to the data to determine the inhibition curve. Maximal inhibition to 0% transport was not always reached, which might be due to endogenous transport present in the vesicular membranes.

**Figure 2:**
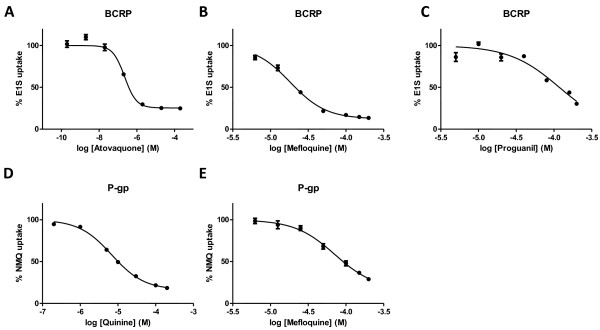
**Concentration-dependent inhibition of potent anti-malarial inhibitors of BCRP and P-gp.** BCRP activity was inhibited according to the dose–response curves for **A** ATO **B** MQ and **C** PG.The inhibition of NMQ transport by P-gp was determined for **D** Q and **E** MQ.

The strongest inhibitory effect for ATO on BCRP-mediated transport was found at median nanomolar range. Transport of estrone sulphate was inhibited with 50% by this compound at 0.23 μM (95% CI 0.17-0.29 μM) (Figure 2A), whereas MQ and PG required the addition of 18 μM (95% CI 17–20 μM) (Figure 2B) and 118 μM (95% CI 93–148 μM) (Figure 2C) to achieve a similar effect on BCRP activity, respectively. Also for the other compound-transporter combinations, IC_50_ values were found in the low to median micromolar range. The effect of Q on P-gp-mediated NMQ transport inhibition was the strongest, and the IC_50_ was defined at 6.8 μM (95% CI 5.9-7.8 μM) (Figure 2D). MQ was a less potent inhibitor with an IC_50_ of 72 μM (95% CI 49–104 μM) (Figure 2E). The inhibitory concentration of ATO and Q transport were within the therapeutic range of blood plasma concentrations after both prophylactic and curative anti-malarial dosing.

## Discussion

In this study, the interaction of anti-malarial compounds CQ, Q, ART, MQ, L, ATO, DHA and PG with the activity of P-gp, BCRP, MRP1-4 and BSEP ABC transport proteins were investigated. ATO was found to be a strong inhibitor of BCRP-mediated transport, which has not been described previously. Also Q was identified as a potent inhibitor of P-gp-mediated transport. In addition, subtle alterations on transporter activity have also been identified for other compound-transporter combinations, both inhibitory and stimulating. These interactions can be either competitive or non-competitive. Allosteric interactions that stimulate transport have been observed for several ABC transporters and are substrate dependent, due to which translation of these results to other transporter-substrate combinations is difficult.

A 50% inhibition of BCRP-mediated transport activity could be achieved with 0.23 μM ATO. This concentration is easily reached in blood plasma during both prophylactic and therapeutic use of ATO, as maximal ATO blood plasma concentrations are around 14 μM (range 8–26 μM) after a daily prophylactic dose of 250 mg [21]. Although the free concentration of ATO is reduced due to its high plasma protein binding, intracellular concentrations at the target site may be higher. ATO is used in a fixed combination with PG in Malarone®, which is prophylactively prescribed to travellers, and at higher dosages to treat falciparum malaria, especially in regions of ACT failure [2, 22]. BCRP is located primarily on the apical side of excretory organs, and it is highly involved in excretion of xenobiotics from the body [23, 24]. As ATO is excreted into bile against steep concentration gradients, involvement of ABC transport proteins such as BCRP is likely [25] and interactions with ATO can occur when elimination of co-administered therapeutics is inhibited.

Indeed, cases of interactions with ATO have been reported. The azithromycin AUC (area under the curve) and maximal concentrations were lower in all patients when taken in combination with ATO by HIV-1 positive children [26]. Although direct interaction of azithromycin with BCRP has not been investigated, interaction at this level cannot be excluded. Moreover, a clear increase in plasma concentration of etravirine, a reverse transcriptase inhibitor, and saquinavir, a protease inhibitor, was observed in a Caucasian female who started malaria prophylaxis with ATO/PG (250/100 mg) fixed dose combination [27]. Etravirine and saquinavir were prescribed to treat HIV1 subtype B in an antiretroviral combination therapy, supplemented with raltegravir and maraviroc. AUCs, during a 12-hour measurement interval, were increased 55% for etravirine and 274% for saquinavir, and peak concentrations after administration of the antiretrovirals was markedly increased. Saquinavir and etravirine have previously been described as potent BCRP inhibitors, but not substrates, with IC_50_ concentrations of 19.5 and 1.0 μM [28, 29]. Both raltegravir and maraviroc do not inhibit BCRP, indicating that interaction with BCRP is specific for saquinavir and etravirine [30]. An alternative or complementary explanation could be interaction at the level of Cytochrome P450 (CYP) enzymes, as PG is mainly metabolized by CYP2C19 but also partly by CYP3A4, saquinavir by CYP3A4 and etravirine mainly by CYP3A4 and to a minor extent by CYP2C9 and CYP2C19 [31–33]. Raltegravir is not metabolized by members of the CYP family, however, maraviroc is a substrate of CYP3A4 [34, 35]. A strong correlation at this level of drug interaction could therefore not be observed, stressing the plausible role of transporter-mediated drug interactions.

Another study demonstrated a significant decrease in ATO plasma concentration when taken in combination with efavirenz, lopinavir/ritonavir or atazanavir/ritonavir therapy [36]. Interaction at the level of metabolism through glucuronidation was proposed. However, as ATO is only marginally glucuronidated but mostly excreted unchanged into the bile, interaction at the level of ABC transport proteins and more specifically BCRP could play an important role [25]. Indeed, efavirenz, lopinavir and atazinavir have been described as inhibitors of BCRP-mediated transport [37]. Lopinavir and efavirenz were found to be stronger inhibitors, and correspondingly, ATO concentration was decreased more drastically in these two combinations compared to atazinavir co-administration.

Other pharmaceuticals that interact with BCRP-mediated transport are fluoroquinolone antibiotics, kinase inhibitors, cytostatics, antifolates, and statins [38–44]. Interactions with ATO therapy might be anticipated when co-administered. These drugs are not widely used in malaria-endemic areas, however, interactions with prophylactic doses of ATO used by travellers can be anticipated.

A 50% inhibition of P-gp-mediated transport by Q was found at a concentration of 6.8 μM. Indeed, in other *in xvitro* cellular uptake experiments Q has been described to be both an inhibitor and a substrate of P-gp [4, 8, 9, 45–48]. The concentration at which Q was effective was lower in the current study than previously described. Most likely this can be attributed to the difference in substrates used. Maximal plasma concentrations reach 30 μM during a seven-day regimen of 10 mg/kg oral dose three times daily of quinine sulphate, and although Q is bound to plasma-proteins to some extent, clinically relevant interactions at the level of P-gp-mediated transport during quinine treatment may be expected [49].

Interactions with Q have been described for ritonavir/lopinavir combination therapy as well as ritonavir monotherapy, and for nevirapine, rifampicin, cyclosporine, and digoxin. Q co-administration with digoxin decreased billary excretion of the latter, indicating specific involvement of transport processes [50]. When co-administered with ritonavir, Q blood plasma concentrations were increased [51]. Ritonavir indeed is both a substrate and inhibitor of P-gp, therefore interaction at this level may explain the increase in Q concentration [52, 53]. After rifampicin, nevirapine and lopinavir co-administration, Q blood plasma concentrations were decreased [49, 54–56]. Rifampicin interacts with P-gp as substrate, inhibitor and inducer, and lopinavir has been found to inhibit P-gp [57–59]. However, this has not been shown for nevirapine. Q is one of the oldest anti-malarial drugs still in use, and although it is not used any more in first-line treatment strategy, its use has increased as it is often applied as an alternative treatment after ACT stock-outs [60]. Furthermore, for treatment of malaria infections in pregnant women it is one of the few compounds that can be applied safely [61]. Adherence to this compound is known to be low due to the large range of common and often plasma concentration-dependent side effects [62]. For these reasons, establishing effective but non-toxic blood plasma concentrations is essential in the treatment of malaria, and interaction with co-administered compounds that mediate P-gp transport should be tightly monitored.

Especially, the interaction of both ATO and Q with antiretroviral medication could have severe implications on treatment strategies for both infections, as HIV is another major contributing factor to morbidity, especially in sub-Saharan regions of Africa [14]. Many different antiretroviral compounds are being prescribed, depending on personal characteristics and resistance status, and for many of these compounds interactions with BCRP have been described.

## Conclusions

Anti-malarial compounds can reduce ABC transporter activity. ATO appeared to be a potent inhibitor of BCRP and Q of P-gp *in vitro*. Both compounds inhibited ABC transporter activity at concentrations equalling prophylactic and effective blood plasma concentrations. Potential involvement in interactions with antiretroviral and antibiotic compounds have been described for ATO and Q, which can be explained by the observed inhibitory effects on BCRP and P-gp transport activity.

## Abbreviations

ABC transporter: ATP binding cassette transporter
P-gp: P-glycoprotein
BCRP: Breast cancer resistance protein
BSEP: Bile salt export protein
MRP: Multidrug resistance-associated protein
ACT: Artemisinin combination therapy
CYP450: Cytochrome P450
IC50: 50% inhibitory concentration
95% CI: 95% confidence interval
HIV: Human immunodeficiency virus
NRT: Nucleoside reverse transcriptase
NNRT: Non-nucleoside reverse transcriptase
PI: Protease inhibitor
CQ: Chloroquine
Q: Quinine
ART: Artemisinin
MQ: Mefloquine
L: Lumefantrine
ATO: Atovaquone
DHA: Dihydroartemisinin
PG: Proguanil.
